# Sex Differences on Mitotane Concentration and Treatment Outcome in Patients with Adrenocortical Carcinoma

**DOI:** 10.3390/life11030266

**Published:** 2021-03-23

**Authors:** Sarah Allegra, Soraya Puglisi, Irene Brescia, Francesco Chiara, Vittoria Basile, Anna Calabrese, Giuseppe Reimondo, Silvia De Francia

**Affiliations:** 1 Laboratory of Clinical Pharmacology “Franco Ghezzo”, Department of Clinical and Biological Sciences, University of Turin, S. Luigi Gonzaga Hospital, 10043 Orbassano, TO, Italy; irene.brescia@edu.unito.it (I.B.); 336124@edu.unito.it (F.C.); silvia.defrancia@unito.it (S.D.F.); 2 Internal Medicine, Department of Clinical and Biological Sciences, University of Turin, S. Luigi Gonzaga Hospital, 10043 Orbassano, TO, Italy; soraya.puglisi@unito.it (S.P.); basile_vittoria@libero.it (V.B.); anna.calabrese@unito.it (A.C.); giuseppe.reimondo@unito.it (G.R.)

**Keywords:** AAC, o,p′-DDD, o,p′-DDE, TDM, gender

## Abstract

(1) Background: In clinical settings, data regarding sex are rarely investigated. In women, factors such as body size and composition, hormonal variations, metabolism, and access to care systems and therapy could strongly influence the pharmacological management and the outcome of the therapy. To underline this sex-related difference, we retrospectively collected data from adrenocortical carcinoma patients treated with mitotane, and then evaluated sex-related pharmacokinetics parameters. (2) Methods: A fully validated chromatographic method was used to quantify mitotane concentration in plasma collected from adult patients, also considering the active metabolite ortho,para,dichlorodiphenylethene (o,p′-DDE). Statistical analyses were used to evaluate the sex influence on drugs pharmacokinetics. (3) Results: We found that sex resulted as predictive factor of plasma mitotane and o,p′-DDE concentrations and significantly influenced the attainment of the therapeutic target of mitotane, implying that female sex could be a risk factor of treatment failure. (4) Conclusions: These results suggest that mitotane therapy should be modulated according to patient sex. Furthermore, the proposed approach could contribute to facilitating and disseminating sex-specific pharmacology.

## 1. Introduction

Adrenocortical carcinoma (ACC) is a rare neoplasm originating in the cortex of the adrenal gland [1]. The prognosis of ACC is unfavorable and surgery remains the only curative treatment [2]. Unfortunately, even in patients undergoing a complete surgical excision, locoregional recurrence or distant metastases occur in most cases [3]. Adjuvant treatment with mitotane is useful to reduce the recurrence rate in high-risk patients [4,5]. Mitotane, alone or in combination with other drugs, is also recommended in metastatic or unresectable diseases [3,6].

Mitotane (ortho,para,dichlorodiphenyl dichloroethane, o,p′-DDD, 2,2-bis[2-chlorophenyl-4-chlorophenyll-l,l-dichloroethane) is an adrenolytic drug derived from the insecticide dichlorodiphenyltrichloroethane, which has been used in treatment centers since 1959 [7]. Mitotane and its two active metabolites, o,p′-DDE (dichlorodiphenylethene) and o,p′-DDA (dichlorodiphenylacetate), have different mechanisms of actions: they act as antineoplastic agents, through the production of free radicals mediating cytotoxicity, and as inhibitors of adrenocortical steroidogenesis, blocking the cholesterol side-chain cleavage enzymes cytochrome P450 (CYP) 11A1 and 11B [8]. Therefore, mitotane and its metabolites can ameliorate signs and symptoms of cortisol excess in secreting ACC [9].

Mitotane therapeutic drug monitoring (TDM) is recommended to optimize the benefit and avoid adverse events [6,10]. Plasma mitotane concentrations between 14 and 20 mg/L are considered as the target range, because concentrations >14 mg/L have been associated with improved outcomes; central neurologic toxicity is more frequent with mitotane concentrations >20 mg/L [11,12,13].

As previously reported, mitotane bioavailability could depend on different factors, such as drug dose, sex, genetics, season in which the drug is administered, and drug-drug interaction [5,14,15,16,17,18].

Sex-related differences in drug pharmacodynamics and pharmacokinetics, response to treatment, and related toxicity have been only partially reported. In a recent study from our group, the female sex was associated with a lower mitotane dose when the drug was used as an adjuvant measure. This is not surprising since data regarding sex-related differences are rarely investigated in clinics [19]. In women, factors such as body size and composition, hormonal variations, metabolism, and access to health care systems and therapy could strongly influence the pharmacological management and treatment outcomes. To this purpose, we described the sex-related differences in a cohort of ACC patients treated with adjuvant mitotane.

## 2. Materials and Methods

### 2.1. Patients and Inclusion Criteria

We performed a retrospective cohort study in ACC patients treated at the S. Luigi Hospital. All patients underwent radical surgery for ACC and then started mitotane as an adjuvant treatment. Inclusion criteria were: age ≥18 years, histologically confirmed diagnosis of ACC according to Weiss score [20], ENSAT stage I–III at diagnosis, complete tumor resection, defined as R0, R1 or RX resection on the basis of surgical and pathologic reports, availability of postoperative follow-up information, and regular quantification of plasma mitotane concentration. Exclusion criteria were: macroscopically incomplete resection, incomplete tumor staging, concomitant cancers within the previous 5 years except for non-melanoma skin cancer treated radically, clinically significant concomitant disease, incomplete follow-up information or follow-up duration of less than 6 months, initiation of mitotane treatment longer than 6 months after surgery, and concomitant postoperative adjuvant therapies (chemotherapy or radiotherapy). Mitotane formulation was Lysodren^®^, 500 mg tablets (Laboratoire HRA Pharma, Paris, France). Mitotane was administered following a low-dose protocol: starting dose of 1 g daily, with increase every 4–7 days, up to 8–10 g daily or the maximum tolerated dose [21]. Collected data were age, sex, date of diagnosis, imaging data, ACC stage, clinical presentation including assessment of hormone secretion, type of surgery, pathology report, adjuvant treatment, date and type of recurrence, treatment of recurrence, and last follow-up or death. Date of diagnosis was defined as the date of surgery and conversion to open adrenalectomy was considered open surgery. Completeness of surgery was established by R status: R0, free resected margins; R1, microscopic involvement of resected margins; RX, not determined, and R2, macroscopic invasion of resected margins. Tumor stage was established according to the ENSAT classification (I–II, confined tumor; III, positive lymph nodes or infiltrating neighboring organs/veins without distant metastases; IV, distant metastases [22]). Biochemical confirmation of hormone excess was requested to categorize an ACC as hormone secreting, information was available only for 238 patients. Patients were stratified for Ki67 index (Ki67 ≤ 10% and Ki67 > 10%). Date of recurrence was defined as the date of radiological evidence of a new lesion. Recurrence-free survival (RFS) was calculated from the time of initial surgery to the first radiological evidence of recurrence (in months). Overall survival (OS) was calculated from the date of initial surgery to the date of death (in months). The study protocol (“Pharmacogenetic determinants mitotane pharmacokinetics”) was approved by the local Ethics Committee. Written informed consent for the study was obtained from each enrolled subject.

### 2.2. HPLC Analysis

Plasma o,p′-DDD and o,p′-DDE concentrations were determined from blood samples obtained at the end of dosing interval, before the next drug dose intake. Considering o,p′-DDE concentrations, the quantification was inserted in the study after a protocol revision; thus, this information was not available for all the enrolled patients.

Patient blood samples were collected in the lithium-heparin tube and centrifuged at 1500 rpm for 10 min at 4 °C. Analytes quantification was performed by a validated high performance liquid chromatography method coupled with UV detection (HPLC-UV) [23]. Substances separation, after specific liquid extraction, was achieved on a RP-C18 column. Internal standard quantification was used, fitted with linear regression.

### 2.3. Statistical Analysis

For descriptive statistics, continuous and non-normal variables were summarized as median values (considering all the enrolled patients) and the interquartile range (IQR, quartile 1; quartile 3) was calculated to measure the statistical dispersion of the data; categorical variables were described as frequency and percentage. All the variables were tested for normality with the Shapiro–Wilk test. The correspondence of each parameter was evaluated with a normal or non-normal distribution through the Kolmogorov–Smirnov test. Non-normal variables were described by median values (plasma concentration). The Mann–Whitney test was used to compare plasma concentration and sex (level of statistical significance *p*-value < 0.05). Any predictive power of the considered variables was finally evaluated through univariate and multivariate linear (β coefficient) for continuous variables, and logistic (OR, odd ratio), considering therapeutic range, regression analyses (IC, interval of confidence at 95%). Factors with *p*-value < 0.2 in univariate analysis were considered in multivariate analysis (level of statistical significance *p*-value < 0.05). Survival curves for RFS and OS were computed according to the Kaplan–Meier method and were compared by means of the log-rank test. All tests were performed with IBM SPSS Statistics 22.0 per Windows (Chicago, IL, USA).

## 3. Results

### 3.1. Study Population

We retrieved data from 246 ACC patients. The o,p′-DDE concentration was determined in 178 samples. Baseline characteristics of our cohort are given in Table 1.

### 3.2. Effect of Sex on Plasma o,p′-DDD Concentrations

**Figure 1:**
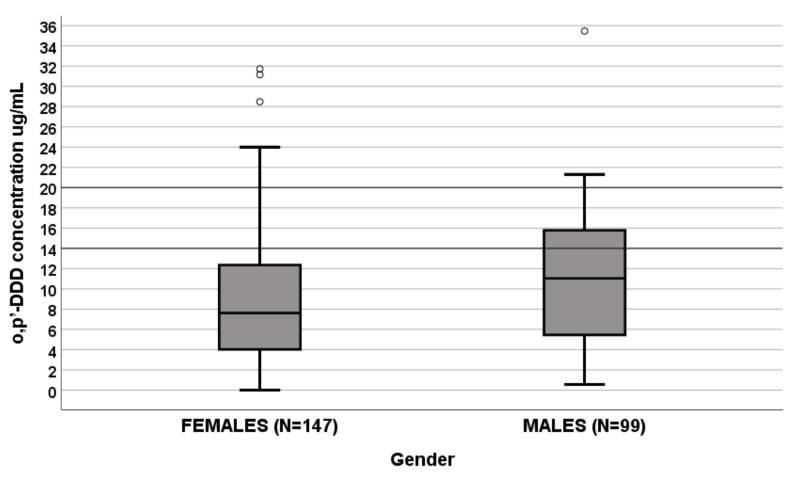
Influence of gender on ortho,para,dichlorodiphenyl dichloroethane (o,p′-DDD) plasma concentrations (μg/mL).

A significant influence of sex on o,p′-DDD levels was apparent since levels were lower in females (N = 147) than males (N = 99) (7.604 μg/mL (IQR 3.971–12.353 μg/mL) vs. 11.029 μg/mL (IQR 5.052–15.779 μg/mL), *p* = 0.007, Figure 1). In a linear regression analysis, sex and hormone secretion were predictive factors (Table 2).

The box plot of gender influence on o,p′-DDD plasma concentrations at the end of dosing interval (µg/mL); boxes and black lines in boxes represent respectively interquartile ranges (IQR) and median values; open dots and stars represent outlier values. Median values (horizontal line), IQR (bars), patient values (black square), highest and lowest value (whiskers), and *p* value are shown.

- Females (N = 147): median o,p′-DDD of 7.604 μg/mL (IQR 3.971–12.353 μg/mL);
- Males (N = 99): median o,p′-DDD 11.029 μg/mL (IQR 5.052–15.779 μg/mL).

### 3.3. Effect of Sex on Plasma o,p′-DDE Concentrations

**Figure 2:**
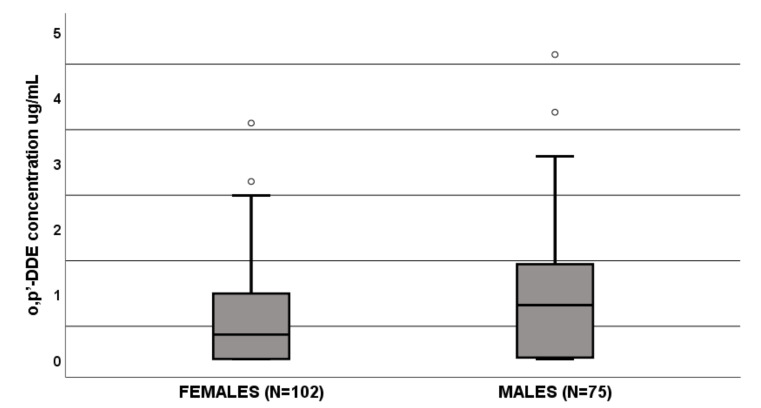
Influence of gender on ortho,para,dichlorodiphenyl dichloroethane (o,p′-DDE) plasma concentrations (μg/mL).

The o,p′-DDE levels were significantly influenced by sex since females (N = 102) had lower values than males (N = 75) (0.373 μg/mL (IQR 0–1 μg/mL) vs. 0.823 μg/mL (IQR 0.21–1.5 μg/mL), *p* = 0.002, Figure 2). In a linear regression, age and sex resulted as predictive factors of o,p′-DDE levels (Table 2). Section 3.1.

The box plot of gender influence on o,p′-DDE plasma concentrations at the end of dosing interval (µg/mL); boxes and black lines in boxes represent respectively interquartile ranges (IQR) and median values; open dots and stars represent outlier values. Median values (horizontal line), IQR (bars), patient values (black square), highest and lowest value (whiskers) and *p* value are shown.

- Females (N = 102): median o,p′-DDE of 0.373 μg/mL (IQR 0–1 μg/mL);
- Males (N = 75): median o,p′-DDE 0.823 μg/mL (IQR 0.21–1.5 μg/mL).

### 3.4. Effect of Sex on Mitotane Concentration Therapeutic Range

**Figure 3:**
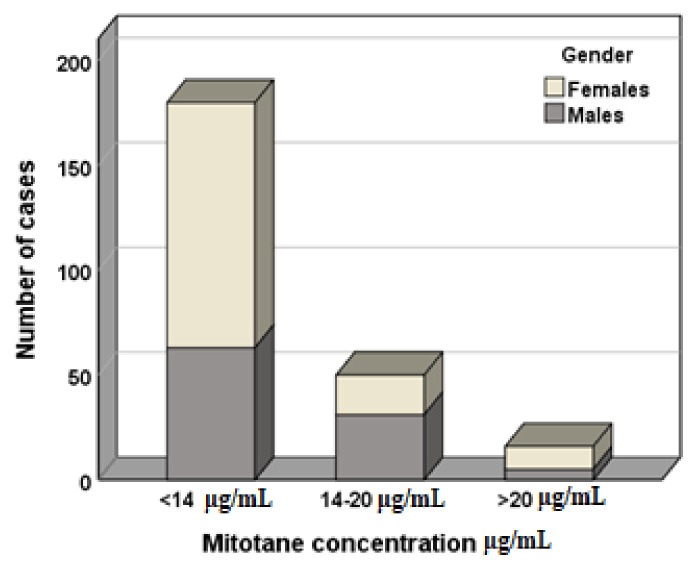
Comparison of gender distribution considering mitotane therapeutic range. <14 μg/mL: females N = 117, males N = 63; 14–20 μg/mL: females N = 19, males N = 31; >20 μg/mL: females N = 11, males N = 5.

In a logistic regression analysis, sex and hormone secretion were mitotane therapeutic range predictive factors (values > 14 μg/mL and <20 μg/mL; Table 3). Figure 3 shows the distribution of males and females based on therapeutic range.

### 3.5. Effect of Sex on Recurrence Free and Overall Survival

**Figure 4:**
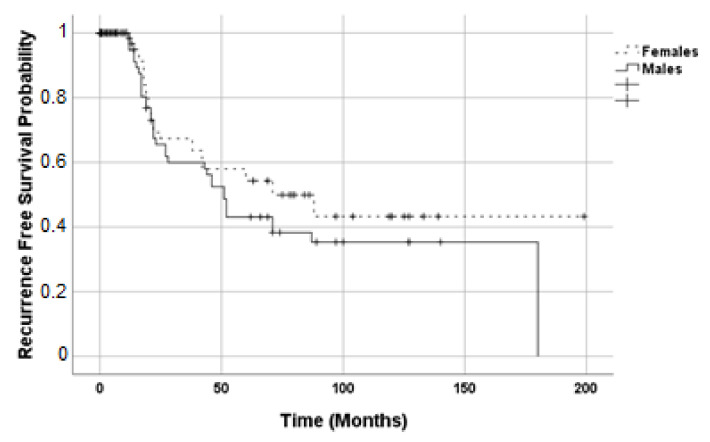
Kaplan–Meier estimates for recurrence-free survival during adjuvant mitotane therapy. Dashed line, female patients (N = 147); solid line, male patients (N = 99).

**Figure 5:**
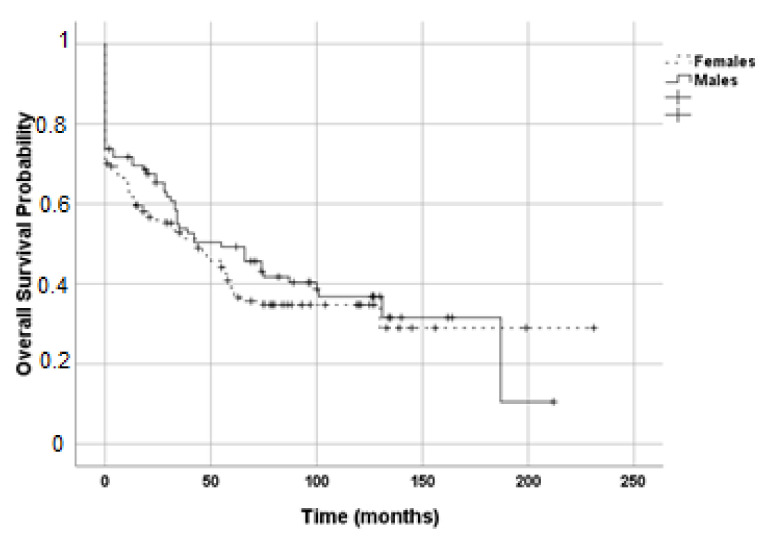
Kaplan–Meier estimates for overall survival during adjuvant mitotane therapy. Dashed line, female patients (N = 147); solid line, male patients (N = 99).

Estimated RFS (*p* = 0.253, Figure 4) and OS (*p* = 0.457, Figure 5) of the two groups by Kaplan–Meier analysis were not different. For RFS, in females, the standard error was 21.448 with a median value of 71 (IC95% 28.90; 113.10); in males, the standard error was 4.146 with a median value of 51 (IC95% 42.87; 59.13). For OS, in females, the standard error was 9.908 and median value of 43 (IC95% 23.58; 62.42); in males, the standard error was 13.946 with a median value of 55 (IC95% 27.67; 82.33).

## 4. Discussion

To date, there is still a large knowledge gap in the field of sex differences in clinical pharmacology. The inclusion of female animals and women in pharmacological studies is still limited, also considering the analysis on safety profile and treatment outcome. However, in the last decade, sex-based significant differences in drug pharmacokinetics and pharmacodynamics have been reported. In particular, males and females differ in drug absorption, distribution, metabolism, clearance, outcome, and toxicity risk [19]. The available evidence suggests that these sex divergences could be due to different factors. For example, hormones influence gastrointestinal motility, thereby drug absorption, and plasma protein levels, altering drug protein binding. During the luteal phase, the high progesterone levels relax the smooth muscle and increase the small intestine retention time, altering drug absorption [19]. The increase of α-1 acid glycoprotein levels alters protein binding and, consequently, free drug concentration, volume of distribution, and half-life [24,25]. Hormone fluctuations throughout the menstrual cycle can also influence hepatic enzyme activity and, thus, drugs metabolism. Progesterone and estrogens, in contrast to androgens, inhibit liver microsomal enzymes. However, progesterone is also a hepatic enzyme activity enhancer [25,26,27]. Eventually, CYP3A4 CYP2A6, and CYP2B6 show a higher activity in women, compared to men; instead, for CYP1A2, CYP2E1, and UDP-glucuronosyltransferase (UGT), a female sex-related activity reduction has been reported [28]. Considering body weight differences, this could influence drug dosage, volume of distribution, and clearance. Moreover, women showed a higher body fat percentage and reduced glomerular filtration rate, affecting drug elimination.

This is the first study that investigated the possible influence of sex on mitotane plasma concentration. We found that sex was as predictive factor of o,p′-DDD and o,p′-DDE concentrations and significantly influenced the achieving of the target plasma mitotane therapeutic range, implying that female sex could be a risk factor for treatment failure. Particularly, we found that more women show mitotane levels under therapeutic range (females N = 117, 80%; males N = 63, 64%) and less female patients reach and maintain mitotane levels in this range (females N = 19, 13%; males N = 31, 31%). Furthermore, more women showed potentially toxic drug levels (females N = 11, 8%; males N = 5, 5%). We hypothesized that this could be due to sex differences in cytochrome P450-mediated metabolism, sexual hormone influence on drug absorption and differences of fat percentage in body composition. However, no difference between females and males has been reported for RFS and OS time with the survival functions. Unfortunately, our research is limited by the lack of data regarding females’ hormonal phase and impedance analysis, not available in a retrospective study. The influence of sex in tumorigenesis and prognosis of ACC has been previously investigated. Keskin and colleagues, in 2013, carried out a study on 24 ACC patients (10 females and 14 males) and observed that sex is a prognostic factor: considering overall survival, males (58 months) had a survival advantage over females (12 months) [29]. With respect to genetic predisposition, both in adults and in the pediatric population, there is a predilection for females [30,31,32]. Recently, Rehane et al. reported a potential sex effect in ACC: mutations in *DGKZ, GOLGA4*, and *NOS3* genes were more frequent in women, with female to male occurrence ratios from 3:1 to 4:0 [33]. In addition, at diagnosis, the mean age was significantly lower in women, possibly due to the consequence of the role of estrogens in adrenal tumorigenesis. The suggested reasons consist in the enhancer role of 17β-estradiol on adrenocortical cell proliferation, in the increased estrogen-related receptor α expression in cancer tissues and insulin-like growth factor 2 stimulation of ACC cell proliferation [34,35,36,37]. Although these studies focused the topic of sex difference in ACC patients, no data were available regarding the impact of sex on plasma mitotane levels. This is a relevant issue, considering the prognostic value of the achieving of the therapeutic mitotane range [11,12,13]. The strengths of our analysis are the large simple size and the monocentric cohort, which offer the advantage to evaluate patients treated with the same protocol of drug administration, thus avoiding a relevant bias.

As is already known, age is one of the best predictors of survival [38,39]: older patients have lower survival rates. In this context, lower active metabolite concentrations have been reported in elderly patients.

We also found reduced o,p′-DDD concentrations and a reduced probability of obtaining therapeutic levels in patients whose tumor had no cortisol secretion. This could be due to the association between hormone secretion and transcriptome signature, as reported in aggressive ACC [40]. Calabrese et al. also observed that secreting tumors were more frequent in younger patients and in women [5], confirming the disadvantage for female ACC patients.

## 5. Conclusions

In conclusion, the results obtained from this retrospective analysis highlight the potential need for sex-specific dosing regimens, to ensure the achievement of therapeutic range. However, no differences in treatment outcome were observed in our cohort. Further and prospective studies, including data about the females’ hormonal phases, fat percentage distribution, lipid parameters, and concomitant drugs, are needed to confirm the role of sex in ACC treatment.

## Figures and Tables

**Table 1:** Demographic, clinical, and pharmacokinetic characteristics of the enrolled patients.

Variable	N = 246
Male, n (%)	147 (59.8)
Female, n (%)	99 (40.2)
Median age, years (IQR)	45.5 (35.2–57.1)
Median body mass index (BMI), kg/m^2^ (IQR)	23.4 (21.6–27.5)
Margins, R status	
R0, n (%)	89 (36.8)
R1, n (%)	57 (23.6)
RX, n (%)	8 (3.3)
Median size, cm (IQR)	7.00 (1.00–12.00)
Median Ki67 index, % (IQR)	25.50 (14.00–40.00)
Median mitotic count (IQR)	12.50 (6.00–25.00)
Tumor stage	
I, n (%)	16 (6.6)
II, n (%)	82 (33.9)
III, n (%)	38 (15.7)
IV, n (%)	28 (11.6)
Hormone secretion	
No secretion, n (%)	73 (30.2)
Secretion, n (%)	82 (33.9)
Cortisol/cortisol + other steroids, n (%)	66 (27.3)
No hormone work up, n (%)	17 (7.0)
Median Weiss score (IQR)	7 (5–8)
Death, n (%)	83 (34.3)
Median recurrence-free survival, months (IQR)	19.00 (7.00–69.00)
Median overall survival, months (IQR)	60.50 (28.25–100.75)
Median o,p′-DDD concentration, µg/mL (IQR)	8.82 (4.36–14.37)
Median o,p′-DDE concentration, µg/mL (IQR) (N = 178)	0.90 (0.48–1.50)
Mitotane therapeutic range	
<14 µg/mL	180 (73.2)
14–20 µg/mL	50 (20.3)
>20 µg/mL	16 (6.5)

N, number; IQR, interquartile range; BMI, body mass index; Ctrough, concentration at the end of dosing interval; %, percentage.

**Table 2:** Factors, in univariate and multivariate linear regression analyses, able to predict o,p′-DDD and o,p′-DDE concentrations.

	o,p′-DDD Concentration		o,p′-DDE Concentration	
	Univariate	Multivariate	Univariate	Multivariate
*FACTOR*	*p* β (IC95%)	*p* β (IC95%)	*P* β (IC95%)	*P* β (IC95%)
*Age*	0.004 −0.185 (−0.147; −0.029)	0.088 −0.123 (−0.123; 0.009)	<0.001 −0.298 (−0.026; −0.009)	0.029 −0.200 (−0.023; −0.001)
*Gender*	0.032 0.137 (0.168; 3.699)	0.010 0.189 (0.644; 4.573)	0.004 0.216 (0.124; 0.629)	0.029 0.199 (0.034; 0.628)
*BMI*	0.616 −0.053 (−0.386; 0.230)		0.978 −0.004 (−0.033; 0.032)	
*Margins*	0.313 −0.081 (−2.632; 0.8499		0.579 −0.050 (−0.342; 0.192)	
*Hormone secretion*	0.003 −0.222 (−2.639; −0.546)	0.005 −0.207 (−2.508; −0.464)	0.711 −0.371 (−0.189; 0.129)	
*Tumor stage*	0.425 0.062 (−0.709; 1.673)		0.143 0.128 (−0.044; 0.301)	0.242 0.114 (−0.079; 0.311)
*Tumor size*	0.655 0.032 (−0.044; 0.070)		0.267 −0.091 (−0.014; 0.004)	
*Weiss score*	0.876 −0.013 (−0.692; 0.590)		0.585 0.052 (−0.076; 0.135)	
*Ki67 index*	0.803 −0.022 (−0.068; 0.053)		0.010 −0.258 (−0.019; −0.003)	0.059 −0.171 (−0.015; 0)
*Mitotic count*	0.630 −0.041 (−0.064; 0.039)		0.089 0.168 (−0.001; 0.015)	0.327 −0.124 (−0.015; 0.005)

*p* value; β: β coefficient; IC95%: interval of confidence at 95%.

**Table 3:** Factors, in univariate and multivariate logistic regression analyses, able to predict mitotane concentration therapeutic range.

	UNIVARIATE	MULTIVARIATE
FACTOR	*p* OR (IC95%)	*p* OR (IC95%)
Age	0.407 0.991 (0.970; 1.012)	
Gender	0.001 0.326 (0.171; 0.619)	<0.001 4.829 (2.156; 10.814)
BMI	0.709 0.977 (0.865; 1.104)	
Margins	0.693 1.154 (0.635; 2.095)	
Hormone secretion	0.001 0.422 (0.249; 0.716)	0.005 0.484 (0.292; 0.801)
Tumour stage	0.100 0.699 (0.455; 1.072)	0.102 0.663 (0.405; 1.085)
Tumour size	0.188 1.011 (0.994; 1.029)	0.728 0.996 (0.976; 1.017)
Weiss score	0.925 0.990 (0.799; 1.227)	
Ki67 index	0.219 0.987 (0.967; 1.008)	
Mitotic count	0.387 0.992 (0.974; 1.010)	

*p*: *p* value; OR: odds ratio; IC95%: interval of confidence at 95%.
